# Introductory Lecture to a Course of Lectures on Materia Medica, Delivered at the Philadelphia Medical Institute

**Published:** 1839-06-08

**Authors:** Joseph Carson


### MEDICAL EXAMINER.

DEVOTED TO MEDICINE, SURGERY, AND THE COLLATERAL SCIENCES.

No. 23.] PHILADELPHIA, SATURDAY, JUNE 8, 1839. [Vol. II.

### INTRODUCTORY LECTURE *to a Course of Lectures on Materia Medica, delivered at the Philadelphia Medical Institute.* By Joseph Carson, M. D.

Gentlemen,—In accordance with the arrangement made for the delivery of the course, that portion of it has been allotted to me, which pertains to the subjects proper of the Materia Medica, or, in other words, the articles employed in the treatment of disease. The *Science of Materia Medica*, as its signification is now received, occupies an extended field, and includes within its range many subsidiary departments. The use of the appellation, in a comprehensive sense, has reasonably been objected to, on account of its being indefinite, or conveying but a partial idea, and I am disposed to accord in the propriety of adopting the name *Pharmacology*, which is more generic in character, and more consistent with modern nomenclature. It comprises all the information possessed with regard to medicinal agents, whether relating to their nature and origin, modes of preparation, or effects and application; hence Materia Medica, Pharmacy, and Therapeutics, are the divisions of which it is susceptible, although these are intimately connected and mutually dependent upon each other.

The department of Therapeutics, however, is not restricted to the articles of the Materia, as in the catalogue containing the whole of them, are not enrolled a number of agents which contribute to the resources of the physician; such are blood-letting in its varieties, electricity and galvanism, hygienic measures, as diet, exercise, &c., which, under appropriate circumstances, are equally efficacious in combatting disease; and this leads me to notice the distinction to be made between the terms *medicine* and *remedy*. In trite language I may state that all medicines are remedies, but it does not follow that the converse is the case, nor indeed is it, for it is to be understood that the former expression has a specific reference to a certain class of bodies, while the latter is general, and applied to agents of every description, which may be beneficially employed. Medicines, then, may be explained to be *substances obtained from nature*, which, from their own inherent qualities, possess the power of affecting the living system, of producing a change or modification in the organic and functional actions, and, if they be deranged, of facilitating their return to a healthy condition.

In the present lecture, I design to lay before you a sketch of the methods available in the study of medicines, considered merely as natural productions, or, as this species of information is called, "a history of simple drugs." The substances composing the Materia Medica, are derived from the three kingdoms of nature, the Animal, Vegetable, and Mineral; they have either contributed to the formation of *organized beings*, or are portions of *unorganized brute masses*, and fall within the distinct province of some one of the several branches of science, which are occupied in the examination of the innumerable and varied works of creation. They are treated of under Botany, Zoology, and Mineralogy, and from these sciences, aided by Chemistry and Physics, is borrowed the light by which we are to be guided in the prosecution of our researches; they form the basis of Pharmacology.

Independent as the sciences mentioned may be supposed to be, at first sight, and difficult as they may appear from the immense number of objects, other than those in which we are interested, recognised by each of them, as legitimately appertaining to it, we should recollect that they were originally the offspring of pharmacological investigation, and, for a long time, were inseparable from Medicine, with which they were incorporated; thus we find that the earliest naturalists were physicians, and Theophrastus and Aristotle may be cited as instances. It has only been within a comparatively recent period, that, facilitated by the advance of civilization, they have been cultivated for their own intrinsic merit, yet continuing to exercise an especial influence upon the Materia Medica, which at all times has been benefited by their extension. Let us take a closer view of the subject, and determine in what manner they are auxiliary.

The first medicines must have been derived from the vegetable kingdom, for as this would attract attention, from its multiplied products, and abundant supply of nutritious sustenance, there would arise a necessity to discriminate between what was wholesome or injurious. Attention once awakened, a knowledge of medicines rapidly advanced, by experimental observation of the effects of vegetable substances, by analogy, and perhaps the habits and instincts of animals afforded no inconsiderable assistance in the prosecution of discoveries. But with all nature before him, and daily progressing in intelligence, the capacity of man could not be restricted to a single field of inquiry; ingenuity aided experience, and all matter, whether vegetable, animal, or mineral, became subservient to his purposes. Confidence in the wisdom and beneficence of Providence stimulated his exertions; he was impressed with the belief, that where misery was imposed, the means of alleviation were only to be sought for to be discovered.

The principle of combination, or separation into groups, appears to be universal. The affinity existing between the species composing certain prominent and easily recognised orders of organic beings, was perceived at an early period, and afforded the data towards the scientific arrangement. From the oldest record of the world, we learn that this separation was made at the beginning; in it we have an account of the creation of "grass and herbs yielding seed," "and the fruit tree yielding fruit," "of moving creatures that have life in the water, of fowl that fly above the earth, and cattle and creeping thing, each of its kind." In proportion as these beings became better known, and their qualities, habits, uses, and modes of life, and propagation understood, the distinctions to be made between such as differed were more clearly defined, and the analogies between such as bore a resemblance more firmly established. Advancing from generals to particulars, the elements of Natural History began to assume form and order. The edifice commenced in remote ages, has gradually been built up by succeeding generations. It is true that the materials have been remodelled, and its aspect changed by the labours of succeeding workmen; but amidst the mutations of time its integrity has been preserved, and it only remains to complete the superstructure, to attract the admiration and veneration of the world.

To comprehend entirely the assistance to be derived from scientific investigation, it will be necessary to consider in succession the different departments of Natural History, and see how far they are adapted to the furtherance of the purposes in view. As the largest proportion of medicines are of vegetable origin, I shall, in the first place, take up Botany.

The study of plants constitutes this science, and in order to render it as complete and accurate as possible, all the particulars relating to them must be attentively examined, and a methodical direction given to researches, otherwise a useless expenditure of toil, with little benefit, would result. Under the latter point of view the utility of Botany is most conspicuous. Although it cannot be expected of us to enter deeply into the science, and to be skilled in all its minutiæ, as this can only be accomplished by those who possess time and taste to pursue it to the full gratification of their curiosity, still some knowledge of its principles will be advantageous. There are several objects to be attained in the prosecution of botanical research. The preliminary step is to recognise plants, and to discriminate between them; they are found to consist of parts, which vary in form, function, and material relations; this then is accomplished by noting the characters of these different parts, and comparing them. To perpetuate and communicate the result of knowledge thus acquired, definitions must be resorted to, indicative of every variety of conformation, and conveying clear ideas of what is intended to be conveyed; a language has therefore been invented, admirably adapted for the purpose intended, in accordance with which, plants are named and described. But this alone is not sufficient to complete the account of the peculiarities; they exhibit among themselves resemblances and discrepancies so evident, and regulated by principles of organization so readily to be detected, that a separation into classes and orders, genera and species, is indispensable to secure a thorough recognition of the alliances which exist between them, and consequently classification, founded upon affinities, furnishes the bond by which the uniformity of the science is preserved, while it confers rank and station, determined by complexity of structure, upon every individual being belonging to the vegetable kingdom.

The advantages derived from the adoption of a comprehensive plan, embracing all the objects of the science, in unfolding which each division has a proper share of importance assigned it, are obvious, when a comparison is instituted between the degrees of improvement presented from time to time, after having been remodelled by the discoveries of the acute and accurate observers, whose lives have been devoted to its cultivation. To the desertion of a partial and contracted method of pursuing inquiries, is to be imputed the fortunate termination of the exertions that have been made to enlarge its limits, and whenever the adaptation of one part to another has been strictly adhered to, they all, collectively, have experienced the benefit, and the whole science has been rendered more profitable in its application. A close correspondence in the manner of *naming*, *describing*, and *classifying* plants, has had the effect of securing to Botany a firm and durable foundation, and this has rigidly been preserved in the works of those who have the highest claims to consideration, as master-builders of the beautiful temple conjointly erected by their industry. The contrast between the botany of the ancients and that of the present age, exemplifies the correctness of these remarks. The former consists of an incomplete account of vegetables, designed to establish and display their medical and sensible properties, without any designation of character, except that of size and habit, with names so inappropriate, or universally applied, and terms of description so vague and loose, as almost to defy the attempt to determine, at subsequent periods, what the originals might have been, while the manner in which they were associated together, was in accordance with actual or fancied powers they possessed upon the functions of life. From these causes, all the information intended to be conveyed was made valueless to succeeding investigators, who were unavoidably coerced to re-construct the science anew, upon principles more solid and lasting, furnished by a faithful examination of all the circumstances calculated to elucidate the elaborate and infinitely variegated modifications of the vegetable world. The systematic labours of Gesner, the Bauhins, Tournefort, and Rivinus, in the 16th and 17th centuries, contributed more to the advancement of the Materia Medica, than did those of all their predecessors, who regarded the study of plants in no other view than as leading to the development of their virtues; for extraordinary as it may appear, the too servile adherence to the very end desired, was the principal reason of failure in accomplishing it.

I shall not detail the peculiarities of the systems erected by these pioneers of reform; it is sufficient to state, that, through their instrumentality, a new way was opened, and many of the impediments removed, and that they were closely followed by others, whose progress has been still greater from the facilities thus afforded, at the head of whom is to be placed the illustrious Sweedish Naturalist, Linnæus, whose profound research and ingenuity will ever entitle him to admiration. The appearance of Linnæus in the domain of Natural History, produced enthusiastic confidence; it created an era in the annals of Botany, which henceforward was destined to assume a position among other positive sciences. Much as the study of the Materia Medica has been promoted by the introduction of the arrangement of this great man, founded upon the sexual organs of plants, it has been equally benefited by the subsequent method of Jussieu, which, from the extension given to it by Decandolle, allows of grouping all vegetables, allied in constitution, habit, and products. The first is known as the *artificial*, the second as the *natural* arrangement. To the latter I shall direct your attention at present.

The primary division of plants is into two classes, *Vascular* and *Cellular*, or those which possess cotyledons, and those which are destitute of them. The first are distinguished by the possession of perfect organs of reproduction or flowers, and, consequently, seed; the second are flowerless, and produce sporæ. Vascular plants are subdivided into *dicotyledonous* and *monocotyledonous;* the former are necessarily composed of cellular tissue, bark, wood, and medulla; the latter consist of cellular tissue, in which the vascular tissue is fixed in bundles, without any distinction of bark, wood, or pith. These characters are not arbitrary and forced; they are of constant occurrence; the existence of one of them is accompanied by that of all the others. Now, before proceeding further in an enumeration of these seemingly uninteresting details, let us see of how much value those already noticed can be to us. You have been told that the *Vascular* or *cotyledonous* plants bore flowers, and were perfect in their organization; these, then, besides pleasing the eye by their elegance and splendour, are also the sources from which are derived luxuriant edible fruit, of so much consequence to our comfort. In contradistinction, the *Cellular*, or *acotyledonous* plants bear no flowers, and are, therefore, imperfect; to these belong the fungi, mosses, and ferns, which are of little value, and afford but few articles as medicines. You have also been informed that the vascular plants were dicotyledonous or monocotyledonous; to the first belong such as contain all the elementary vegetable substances, except gluten; from them are obtained the most elaborate products of vegetation, as the fixed and volatile oils, acids, camphor, the resins, &c. To the second appertain those which abound in fecula, and whose epidermis contains a large amount of silex; generally, their fruit is devoid of fixed oil, for which, apparently, gluten is substituted; by them the most wholesome nutriment is afforded, and for this purpose many of them are cultivated; such are the various species of grain, arrow-root, sago, &c.

But to reduce our classification to *orders*. It is here that the beauty and utility of the system, founded upon affinities, display themselves. For the purpose of illustration, I shall select such facts as will readily strike your comprehension, presented by the very natural order or family *Cruciferæ*. The flowers, and mode of producing the seed, in the individuals composing this family, are similar, so as to render them distinguishable at a glance; they universally possess stimulating, pungent, and acrid properties, which appertain to a volatile substance; they contain a large proportion of azote; hence their purgency has been attributed to ammonia. The putrefaction of cruciferous plants is attended with a peculiar exhalation, resembling that from animal matter; and the facility with which they undergo decomposition, is a further evidence of an analogy, which arises from the presence of azote in both. From their stimulating qualities they can be rendered valuable auxiliaries in medicine, and mustard may be cited as an instance. We are familiar with cabbage, turnip, horse-radish, and the different species of cress, which are eminently anti-scorbutic, and from the combination of mucilage and saccharine principle, are exceedingly nutritious.

The order *Rosaceæ*, which corresponds to the class *Icosandria*, of Linnæus, is characterized by the astringency pervading the several parts of the plants belonging to it; they owe this to the existence of *tannin;* as types, we may refer to the species of Rubus and Potentilla. Those which produce fruit, afford such as is most grateful to the palate,—as the apple, pear, strawberry, and raspberry,—the flavour of which is attributable to malic acid. Those which belong to the *Amygdaleæ*, or almond tribe, as the peach, plumb, cherry, &c., contain the element of prussic acid, *amygdalin*, which when existing in quantity renders them deleterious; but from the small amount usually present, renders the flavour agreeable.

I might, in this cursory manner, go through the whole catalogue of orders, but this is unnecessary, inasmuch as what has been adduced is sufficient to give an idea of affinities, and to support us in maintaining the advantage which accrues from tracing them. It cannot be withheld, however, that many difficulties and objections lie in the way of the extension of this method, and of its applicability to practical purposes; exceptions occasionally arise to startle the too enthusiastic believer in an exclusive system, and his faith in the stability of analogous characteristics is so weakened, as to induce him to deny that they are worthy of any attention. It is therefore important to examine these opposing facts, and I shall first notice such as are met with in the order *Umbelliferæ*. The plants belonging to this order are exactly similar in structure, and for the most part possess analogous properties, which differ nevertheless in the different parts. Thus the seeds are stimulating and aromatic, while the herbaceous portions are usually poisonous, and always to be suspected; the viscid concrete juices of a number of them possess anti-spasmodic properties, as assafœtida, gum ammoniac, and galbanum. But from no other order could be selected individuals having more opposite characters; for example, the several species of Cicuta are highly deleterious and narcotic, while the cellery, the parsnip, and carrot are esculent. To reconcile these apparent discrepancies, we must take into consideration the effects of cultivation, soil, climate, and other physical local causes, which modify and destroy unwholesome qualities. The vegetables mentioned are in their wild condition far from being what they are when used as articles of food. None among the plants belonging to this family exhibit a capability of change more than *Conium maculatum*, or hemlock. If reared in a mild southern climate, it retains all the virulence for which it is remarkable, but if transplanted to one less genial, it loses this property and becomes inactive. It is stated that in the Crimea, even this plant is used by the peasants as food.

The order *Solaneæ* embraces also a variety of plants, which are manifestly opposite in properties. From the potato, tomato, and egg plant are obtained nutriment; the capsicum possesses a stimulating principle; and the dulcamara, nightshade, and henbane are highly narcotic. To all of them belong disagreeable qualities, rendering most of those which are esculent unfit for use without preparation, and with regard to the degree or amount in which these qualities exist, as in the previous family, the same effect of modifying and destroying them is produced by tillage.

The occurrence of many irreconcileable facts, moreover, is to be attributed to the want of knowledge, which may, at some future period, be satisfactorily settled. Admitting, then, that this system is not perfect, of what advantage can it be to the Materia Medica, to prosecute the study of it? In reply, I assert, that sufficient is known and determined to afford immense facilities in methodising researches and rendering them practical, and the very obstacles themselves are useful by awakening our energies to the endeavour to trace out the causes of disagreement. Yet it may be supposed, that the advantages of Botany are remote, and that it exercises no especial influence upon the Materia Medica; a few words will suffice to determine this point, and to show to what extent the science is immediately serviceable, and why some degree of acquaintance with it should be possessed. The substitution of the simple and chaste nomenclature of Botany, where vegetable productions are concerned, for the obscure and perplexing names formerly used, is among the greatest improvements of which our latest standard authorities can boast, and for this our own classical Pharmacopœia is most distinguished; besides, the language of Botany has been introduced into every work upon the Materia Medica, and the pages of the text books from which rudimental instruction is derived, are replete with its descriptions, without possessing the key to which, they must remain as sealed and unintelligible as the Sybiline leaves, or an hieroglyphical inscription.

Again, we have daily presented to our inspection, as medicinal substances, roots, leaves, seeds, &c., and is it not of importance to discriminate between them? To identify them? To detect adulterations of them? To ascertain whether the introduced matters are innocent or the reverse, and thereby preserve our reputation from injury occasioned by the unfortunate results of their administration? And who will be best qualified to accomplish all this, except he who understands the sources from which medicines are procured, and the discriminating peculiarities by which they are known? Will he who is skilled in botanical characters be at a loss to determine the place of each, when he has before him seeds appertaining to two distinct families?

The substitution of one article for another, and the introduction of new medicines will be materially aided by the same information. We are constantly in the habit of acting upon the principles which have been but partially presented to you; then, why not render them more efficient by more thorough acquaintance with them?

From the animal kingdom are derived a number of articles which are highly useful in medicine. Animals are ranged under four heads, viz. *Vertebral*, *Moluscous*, *Articulated*, and *Zoophites*. The vertebral are most perfect and include such as are the conspicuous inhabitants of earth, air, and water; they are subdivided into *Viviparous* and *Oviparous*. At the head of the first is man; to this division belong also the whale yielding spermaceti, the musk deer, and castor; within the latter are found the species of sturgeon, which furnish Icthycolla, the species of birds of which the eggs are in use, and several kinds of reptiles formerly in vogue. To the Moluscous family appertain *Sepia officinalis*, and the oyster from which is made the *Testa præparata* of the Pharmacopœia.

Among articulated animals are placed leeches, which, although not included in the Materia Medica, are indispensable to the physician. One of the divisions, the Crustaceæ, derives its name from the hard resisting case covering the animals; it is composed of phosphate of lime. Another division of this family is appropriated to Insects: this is worthy of consideration; here the blistering fly and its varieties, cochineal, honey, and wax, attract our notice.

Zoophites are of little consequence; as examples of their productions, sponge and coral may be mentioned.

To the mineral kingdom we are indebted for some of the most energetic medicines; but the details to be presented are so intimately connected with chemistry and physics, as to render it difficult to separate them; the best plan, perhaps, is to consider them conjointly. Our whole knowledge of mineral substances, as, for instance, metals and earths, is based upon their chemical attributes, and physical characters, and from them are deduced their affinities. These two maintain a certain relation to each other: thus, when substances of this denomination are subjected to the operation of the laws of Chemistry, and undergo consequent changes, these are attended with alteration in their physical properties. Hence, both must furnish the data upon which to found a classification. To illustrate this, let us take one or two examples. Iron possesses the physical characters of a metal, as it is hard, heavy, malleable, ductile, and shining; Chemistry determines it to be a simple electro-positive substance, and capable of union with oxygen; by this union, its physical peculiarities as a metal are lost, new ones are acquired as an oxide, which can combine with acids and form salts, it is therefore *Basifiable*. Lime possesses the physical properties of an earth; Chemistry shows that it is an alkaline earth; it can be decomposed into a metal (calcium) and oxygen; it is capable of uniting with acids, constituting a base. In the first instance, iron belongs to the class of *electro-positive bodies;* sub-class *Metals;* order *Basifiable;* division, *those forming ordinary oxides.* Lime is ranged under all these heads except the last, as it belongs to the division of *Earths.* Now, *à priori*, would it have been possible to have conjectured that there could be any relation between lime and a metallic body? Yet classification, founded upon chemical analysis, associates it with the oxydized metals.

In addition to the facilities which Chemistry affords to nomenclature and classification, which implies an exact knowledge of the nature of mineral substances and their similitudes, we must regard its utility in another point of view. Familiarity with it obviates the embarrassment which would perpetually occur in the administration of medicines; it would be useless to cite instances corroborative of this assertion; the admixture of conflicting matters would set all rule and precision at defiance, were the ingredients of compounds not carefully selected with reference to their compatability, and the results expected from their exhibition would be defeated. Chemistry, moreover, exercises immense agency in imparting greater efficiency to articles supplied from all the sources that have been adverted to; it produces from those apparently inert others that are highly powerful—it separates active ingredients from matter that is inefficient and useless, and it developes principles which are otherwise hidden from observation. By the isolation of such principles and fitting them for practical purposes, Chemistry has benefited mankind more than all the branches of science contributing to the Materia Medica combined; and the daily announcement of its inexhaustible resources, exhibits ample evidence of the indefatigable industry of those individuals who are labouring to complete its triumphs. To us it is all important; the attention cannot be directed to any quarter where proof does not exist of its utility. It is the touchstone by which fraud is discoverable, and the sure safeguard against dishonesty.

Before dismissing the subject of the means of obtaining a knowledge of medicines, let me notice more particularly that founded upon their sensible qualities. These to a certain extent will serve us, if the articles submitted to this test be familiar, and essentially differ in the impression made upon the senses. But all sensible qualities are fallacious, inasmuch as they have their existence in organs which vary in nicety of discrimination, and for which no standard can be determined. The impression on the sight, taste, touch, and smell differs in each individual; consequently no language can express the infinite shades of disparity; and further, there are substances which are so analogous in sensible properties, that the greatest accuracy cannot detect a difference, yet they may be so opposite in their effects upon the animal economy, as to render a substitution of one for another extremely serious in its consequences; as instances, the several vegetable alkaloids may be cited. Should the resort of chemical reagents fail, I am acquainted with no method of discrimination. Perhaps the impetus that has been given to researches into the form, the magnitude, and the arrangement of particles, may afford the means of clearly demonstrating what hitherto has resisted the best directed efforts.

I have now passed in review the several departments of science from which are furnished the elements of Pharmacology; they are mutually allied and connected together, supported and sustained by the influence that one has upon the other, they present a whole of firm and durable materials unaffected by the fluctuation of opinion. New investigations and more enlarged experience will continue to consolidate them.

Finally, to complete the account of simple drugs, it is essential to indicate the localities whence they are procured, to give their commercial history, as well as the modes of collection and preparation for the market. Under these heads are included a number of circumstances which are interesting and important, and which will be detailed when the different substances become especial objects of attention.

